# Abieslactone Induces Cell Cycle Arrest and Apoptosis in Human Hepatocellular Carcinomas through the Mitochondrial Pathway and the Generation of Reactive Oxygen Species

**DOI:** 10.1371/journal.pone.0115151

**Published:** 2014-12-11

**Authors:** Guo-Wei Wang, Chao Lv, Zhi-Ran Shi, Ren-Tao Zeng, Xue-Yun Dong, Wei-Dong Zhang, Run-Hui Liu, Lei Shan, Yun-Heng Shen

**Affiliations:** 1 School of Pharmacy, Shanghai Jiao Tong University, Shanghai 200240, PR China; 2 School of Pharmacy, Second Military Medical University, Shanghai 200433, PR China; 3 School of Pharmacy, Fujian University of Traditional Chinese Medicine, Fujian 350108, PR China; Institute of Biochemistry and Biotechnology, Taiwan

## Abstract

Abieslactone is a triterpenoid lactone isolated from *Abies* plants. Previous studies have demonstrated that its derivative abiesenonic acid methyl ester possesses anti-tumor-promoting activity *in vitro* and *in vivo*. In the present study, cell viability assay demonstrated that abieslactone had selective cytotoxicity against human hepatoma cell lines. Immunostaining experiments revealed that abieslactone induced HepG2 and SMMC7721 cell apoptosis. Flow cytometry and western blot analysis showed that the apoptosis was associated with cell cycle arrest during the G_1_ phase, up-regulation of p53 and p21, and down-regulation of CDK2 and cyclin D1. Furthermore, our results revealed that induction of apoptosis through a mitochondrial pathway led to upregulation of Bax, down-regulation of Bcl-2, mitochondrial release of cytochrome c, reduction of mitochondrial membrane potential (MMP), and activation of caspase cascades (Casp-9 and -3). Activation of caspase cascades also resulted in the cleavage of PARP fragment. Involvement of the caspase apoptosis pathway was confirmed using caspase inhibitor Z-VAD-FMK pretreatment. Recent studies have shown that ROS is upstream of Akt signal in mitochondria-mediated hepatoma cell apoptosis. Our results showed that the accumulation of ROS was detected in HepG2 cells when treated with abieslactone, and ROS scavenger partly blocked the effects of abieslactone-induced HepG2 cell death. In addition, inactivation of total and phosphorylated Akt activities was found to be involved in abieslactone-induced HepG2 cell apoptosis. Therefore, our findings suggested that abieslactone induced G_1_ cell cycle arrest and caspase-dependent apoptosis via the mitochondrial pathway and the ROS/Akt pathway in HepG2 cells.

## Introduction

Hepatocellular carcinoma, also called malignant hepatoma, is the fifth common cancer in human and the third leading cause of cancer death worldwide, responsible for over 600,000 deaths every year [1]. Clinically, the only medical treatments for hepatocellular carcinoma are surgical resection and liver transplantation in patients [2]. Unfortunately, owing to high recurrence rate after resection, most patients are not eligible for surgery [3]. Conventional chemotherapeutic and radiotherapeutic treatments have led to serious health problems, as they can kill healthy cells as well. Moreover, resistance to chemotherapy is frequently observed. Therefore, developing novel efficient drugs with minimal side effect and understanding their molecular mechanisms are necessary for improving hepatocellular carcinoma therapy.

Apoptosis, a process of programed cell death, is the most common mechanism exploited by targeted chemotherapies that can induce death in cancer cells [4]. Apoptosis is characterized by distinct morphological changes, including membrane blebbing, cell shrinkage, loss of mitochondrial membrane potential (MMP), chromatin condensation and DNA fragmentation [5]. There are two established pathways that result in apoptosis: the extrinsic cell death pathway (cell death receptor pathway) and the intrinsic cell death pathway (the mitochondria-initiated pathway) [6]. At the biochemical level, apoptosis is mediated by the activation of a class of cysteine proteases known as caspases [7]. Caspase activation mainly occurs *via* death receptor pathway activation or mitochondrial membrane depolarization. Mitochondrial-dependent apoptosis is regulated principally by the Bcl-2 protein family. Bax is a cardinal proapoptotic member of Bcl-2 family proteins, which regulates the critical balance between cell survival and death [8]. In response to apoptotic signals, Bax transforms into a lethal mitochondrial oligomer and becomes activated to cause mitochondrial damage, a key step for the intrinsic pathway to apoptosis [9], [10].

Reactive oxygen species (ROS) is a collective term embracing a variety of oxygen-containing, reactive, and short-lived molecules. ROS are the byproducts of aerobic respiration and primarily arise from the mitochondria [11], [12]. It has become increasingly evident that certain anticancer agents induce intracellular ROS that is either the primary mechanism of cell death or is a secondary indirect effect that may lead to cell death [13], [14]. At low concentrations, ROS has been identified as a second messenger in signaling pathways. However, high levels of ROS in mitochondria may cause mitochondrial membrane depolarization, release of mitochondrial factors and triggering of caspase cascades [15]. Previous reports have shown that ROS acts upstream of mitochondria-mediated apoptosis by promoting Bax translocation to mitochondria [16]–[18], activating JNK activity [19], or repressing Akt and NF-kB activity [20], [21]. Therefore, ROS play a key role in mitochondria-mediated apoptosis.

Plants are considered to be one of the most important sources of anticancer agents. Plant-derived natural products (such as taxol [22], curcumin [23], and tetrandrine [21], [24]), that can activate cell apoptosis, have great potential in cancer therapy. Abieslactone, previously reported from the bark and leaves of *A. mariesi* in 1965 [25], is a natural triterpenoid lactone that we recently isolated from the branches and leaves of *A. faxoniana*. It has been reported that its derivative abiesenonic acid methyl ester could suppress tumor promoter-induced phenomena *in vitro* and *in vivo* [26]. In this study, we demonstrated that abieslactone inhibited the growth and proliferation of three human hepatoma cell lines (HepG2, SMMC7721, and Huh7) but had low cytotoxicity to normal hepatic cells (QSG7701). HepG2 and SMMC7721 cells were more sensitive to abieslactone treatment than Huh7 cells. We further investigated its mechanism of action using HepG2 and SMMC7721 as representative cell line models. Although abieslactone could induce cell cycle arrest and apoptosis in liver cancer cells HepG2 and SMMC7721, the molecular mechanisms in two cell lines are not the same. Abieslactone induced cell cycle arrest at G_1_ phase and caspase-dependent apoptosis *via* both mitochondrial pathway and the ROS/Akt pathway in HepG2 cells, but the ROS/Akt pathway was not involved in abieslactone-induced SMMC7721 cells apoptosis.

## Materials and Methods

### Drugs and antibodies

Abieslactone was isolated from the branches and leaves of *A*. *faxoniana* (purity>98% as determined by analytical HPLC). Propidium iodide (PI), Hoechst 33258, dimethylsulfoxide (DMSO), [3-(4,5-dimethylthiazol-2-yl)-2,5-diphenyltetrazolium bromide] (MTT), Z-VAD-FMK, N-acetyl-L-cysteine (NAC), doxorubicin (DOX), Dulbecco's Modified Eagle's Medium (DMEM), fetal bovine serum (FBS), phosphate buffered saline (PBS), RNase A, penicillin and streptomycin were purchased from Sigma Chemical Co. (St. Louis, MO, USA). Rhodamine 123 and DCFH-DA were purchased from Eugene Co. (OR, USA). The annexin V-FITC apoptosis detection kit was purchased from Beyotime Institute of Biotechnology (Shanghai, China). Mouse polyclonal anti-human Bcl-2, rabbit polyclonal anti-human Bax, cytochrome c, p53, p21, cyclin D1, CDK2, caspase-3, caspase-9, PARP, p-Akt, Akt and NF-kB p65 antibodies were purchased from Cell Signaling Technology (Beverly, MA, USA). Antibodies specific to β-actin and horseradish peroxidase-conjugated secondary antibodies (goat-anti-rabbit, goat-anti-mouse) were purchased from Santa Cruz Biotechnology (Santa Cruz, CA, USA).

### Cell lines and cell culture

The human hepatomacell lines (HepG2, SMMC7721, and Huh7) as well as the normal cell lines (QSG7701) were obtained from Shanghai Institute of Materia Medica, Chinese Academy of Sciences. The cells were grown in plastic culture flasks under standard conditions (37°C with 5% CO_2_ in a completely humidified atmosphere) using DMEM medium supplemented with 10% heat-inactivated FBS, 2 mM L-glutamine, 100 units/mL penicillin and 100 µg/mL streptomycin.

### Cell viability assay

**Figure 1:**
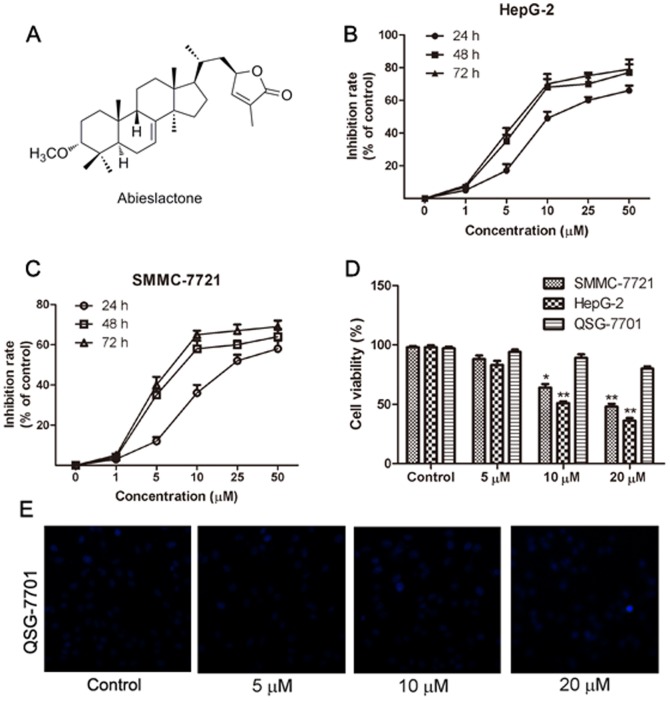
The chemical structure of abieslactone and its growth-inhibiting effect on HepG2, SMMC7721 and QSG7701 cells. (A) The chemical structure of abieslactone. (B and C) Viability of HepG2 and SMMC7721 cells after exposure to 0.1% DMSO or various concentrations of abieslactone for 24, 48, and 72 h. The data are expressed as the means ± SEM of three independent experiments. (D) Cell viability after 5, 10, and 20 µM abieslactone treatment in HepG2, SMMC7721 and QSG7701 cells for 24 h. The results are the mean ± SEM from three independent experiments. *P<0.05; **P<0.01 *vs*. the untreated control. (E) The morphological nuclear changes in QSG7701 cells treated with abieslactone at different concentrations (5, 10, and 20 µM). The cells were stained with Hoechst 33258 for 30 min in the dark to examine the cleaved nuclei, which is a sign of apoptosis.

Cell viability was determined by the MTT assay. Briefly, cells were seeded in 96-well plates at 6×10^3^ cells/well and were treated with abieslactone (0, 1, 5, 10, 25, 50 µM) for various time periods (24, 48, 72 h) [27]. Doxorubicin (0, 0.25, 0.5, 1, 2.5, 5, 10 µM) was used as a positive control in this experiment. Cultures were also treated with (0.1%) DMSO as the untreated control. After treatment, 10 µL of MTT solution (5 mg/mL) was added to each well and the plates were incubated for 2–4 h at 37°C. The supernatant was then removed from formazan crystals and 100 µL of DMSO was added to each well. The absorbance at 570 nm was read using an OPTImax microplate reader. The cell viability was calculated by dividing the mean optical density (OD) of compound-containing wells by that of DMSO-control wells. Three separate experiments were accomplished to determine the IC_50_ values. As shown in **Fig. 1B** and **C**, a clear dose-dependent cell death was observed after the cells were treated with abieslactone for 24 h. Thus, 24 hours was the preferred time period of choice for the rest of the experiments.

### DNA fragmentation assay

Cells were treated with 5, 10 or 20 µM abieslactone for 24 h. DNA fragmentation was measured using Hoechst 33258 staining [28]. The cells were fixed with 4% paraformaldehyde for 15 min at room temperature. Then, washing with PBS, the cells were stained with Hoechst 33258 (50 µg/mL) at 37°C in the dark for 30 min. The cells were washed and resuspended in PBS to assess nuclear morphology under fluorescence microscopy.

### Cell apoptosis assay

The apoptotic cells were quantified using the annexin V and PI double staining kit [28]. Briefly, cells were treated with abieslactone (5, 10 and 20 µM) for 24 h. After treatment, the cells were collected, washed with PBS, and resuspended in 200 µL binding buffer containing 5 µL annexin V (10 µg/mL) for 10 min in the dark. The cells were then incubated with 10 µL PI (20 µg/mL), and the samples were immediately analyzed using flow cytometry. For the caspase inhibitor analysis, the cells were pretreated with 20 µM Z-VAD-FMK for 2 h and then incubated with 20 µM abieslactone.

### Cell cycle assay

The DNA content of cells in the G_0_/G_1_, S and G_2_/M phases were measured using flow cytometry [27], [28]. Cells were incubated with abieslactone (5, 10 and 20 µM) for 24 h. After treatment, the cells were collected, washed with PBS containing 2% FBS. 3×10^5^ cells/mL were fixed with cold absolute ethanol overnight at 4°C in a 15 mL polypropylene and V-bottomed tube. After washing with PBS twice, the cells were incubated with 1 mL PI staining solution (3.8 mM sodium citrate, 50 µg/mL PI in PBS). Add 50 µL of RNase A solution (100 µg/mL RNase A) and incubated for 30 min at room temperature in the dark. The DNA content of cells and cell-cycle distribution were analyzed by flow cytometry.

### Mitochondrial membrane potential measurement

The mitochondrial membrane potential (MMP) was measured by flow cytometry after staining liver cancer cells with Rhodamine 123 (Rh123), a cationic lipophilic fluorochrome [28]. The Rh123 uptake by the mitochondria is proportional to the MMP. Briefly, cells were treated with abieslactone (5, 10 and 20 µM) for 24 h and were then incubated with Rh123 at a final concentration of 10 µM for 30 min at room temperature in the dark. After being washed twice with PBS, cells were resuspended in 1000 µL PBS and analyzed using flow cytometry with excitation and emission wavelengths of 488 and 530 nm, respectively.

### Intracellular ROS production measurement

ROS levels were detected using a flow cytometer and a microplate spectrophotometer (Molecular Devices, Sunnyvale, CA, USA) [20]. Cells pretreated with 10 mM ROS scavenger NAC for 1 h were incubated with 20 µM abieslactone for 24 h. Cellular viability was determined in the presence of 10 mM NAC for 1 h. After treatment, cells were harvested and washed with PBS and suspended in DMEM containing 10 µM 5(6)-carboxy-2′,7′-dichlorodihydrofluorescein diacetate (carboxy-H_2_DCFDA; Invitrogen) at 37°C for 20 min. The cells were then washed twice with PBS and subjected to flow cytometry analysis.

### Western blot analysis

Cells were treated with abieslactone for 24 h, washed twice with PBS, and lysed for 30 min on ice using WIP cell lysis reagent (150 mM NaCl, 50 mM Tris, pH 8.0, 1% Triton X-100, 1 mM Na_2_EDTA, 1 mM EGTA, 2.5 mM sodium pyrophosphate, 1 mM β-glycerophosphate, 1 mM Na_3_VO_4_ and 1 µg/mL leupeptin) [28]. The insoluble protein lysate was removed by centrifugation at 12000 rpm for 15 min at 4°C [21]. The protein concentrations were determined using a NanoDrop 1000 spectrophotometer (Thermo Scientific, USA). Proteins were electrophoresed using 15% SDS-PAGE and transferred to a PVDF membrane. After blocking with 5% (w/v) non-fat milk and washing with Tris-buffered saline-Tween solution (TBST), the membranes were incubated overnight at 4°C with specific primary antibodies (1∶1000) and anti-rabbit IgG (1∶2000) or anti-mouse IgG (1∶2000) secondary antibodies for 1 h at room temperature and then washed again three times in TBST buffer. The membrane was incubated with enhanced chemiluminescence substrate solution (Santa Cruz Biotechnology, Inc.) for 5 min according to the manufacturer's instructions and visualised with autoradiography film.

### Statistical analysis

Results were expressed as mean ± SEM. Data were analyzed by one-way analysis of variance (ANOVA) followed by the Dunnett's test. A value of *P*<0.05 was considered significant.

## Results

### Abieslactone exhibits selective cytotoxicity toward tumor cells

The hepatoma cell lines (HepG2, SMMC7721, and Huh7) and normal hepatic cell line (QSG7701) were used to assess the cytotoxic effects of abieslactone (chemical structure shown in **Fig. 1A**). Doxorubicin was employed as a positive control in this experiment, with IC_50_ values of 0.5, 0.3, 0.7 and 2.9 µM, respectively. In the three hepatoma cell lines, HepG2 and SMMC7721 cells were more sensitive to abieslactone treatment than Huh7 cells; the 50% inhibitory concentrations of cell viability (IC_50_) were determined to be 9.8 µM (HepG2), 14.3 µM (SMMC7721) and 17.2 µM (Huh7). Interestingly, abieslactone demonstrated low toxicity to normal hepatic cells (QSG7701, IC_50_>50 µM).

We also confirmed the inhibition effect of abieslactone against hepatoma cell growth by analyzing the percentages of living and dead cells. The number of living and dead cells was measured using a cell viability analyzer. The MTT assay showed that abieslactone inhibited HepG2 and SMMC7721 cells growth in a dose and time dependent manner (**Fig. 1B** and **C**). **Fig. 1D** showed the survival ratio of hepatoma cells (HepG2 and SMMC7721) and normal hepatic cells (QSG7701) treated with 5, 10 or 20 µM abieslactone for 24 h. Compared to the untreated control, the number of living cells in abieslactone-treated groups was significantly decreased in a dose-dependent manner. The normal hepatic cells QSG7701 were less sensitive to the inhibitory effects of abieslactone than those of HepG2 and SMMC7721 cells, suggesting that abieslactone has tumor cell selectivity. We also tested morphological nuclear changes in QSG7701 cells using Hoechst 33258 staining (**Fig. 1E**). QSG7701 cells showed less nuclear change after being treated with abieslactone at different concentrations.

### Abieslactone induces cell apoptosis

**Figure 2:**
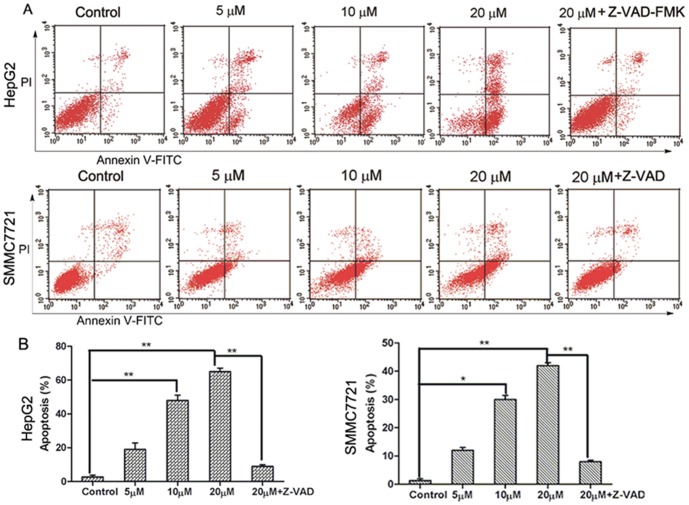
Abieslactone-induced apoptosis in HepG2 and SMMC7721 cells. (A) Apoptosis was evaluated using an annexin V-FITC apoptosis detection kit and flow cytometry. The X- and Y-axes represent annexin V-FITC staining and PI, respectively. The representative pictures are from HepG2 and SMMC7721 cells incubated with different concentrations of abieslactone or caspase inhibitor (Z-VAD-FMK 20 µM). (B) Abieslactone induced apoptosis in HepG2 and SMMC7721 cells in a dose dependent manner. Z-VAD-FMK markedly reduced apoptosis in HepG2 and SMMC7721 cells treated with high-dose abieslactone. The data are expressed as the means ± SEM of three independent experiments with the similar results. *P<0.05; **P<0.01 *vs*. the untreated control.

First, we determined whether abieslactone-induced cell death was caused by apoptosis. Cell apoptosis was revealed by Annexin V-FITC/PI staining in HepG2 and SMMC7721 cells treated with abieslactone at different concentrations, though SMMC7721 cells were more resistant to abieslactone treatment (**Fig. 2**). 24 h after 20 µM abieslactone treatment, most HepG2 cells were undergoing apoptosis, whereas SMMC7721 cells showed less apoptosis after being treated with 20 µM abieslactone for 24 h. Furthermore, no significant early or late apoptosis was observed in HepG2 and SMMC7721 cells pretreated with Z-VAD-FMK, an extensive caspase inhibitor (**Fig. 2**). These results suggested that abieslactone induced HepG2 and SMMC7721 cell apoptosis possibly *via* the caspase pathway.

**Figure 3:**
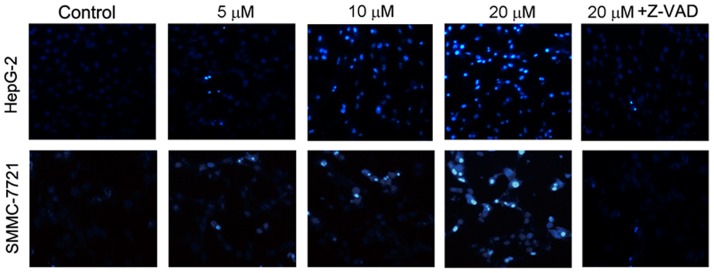
Nuclear morphology of HepG2 and SMMC7721 cells treated with 5, 10, and 20 µM abieslactone or 20 µM Z-VAD-FMK for 24 h was determined by staining with Hoechst 33258.

To further verify abieslactone-induced apoptosis in HepG2 and SMMC7721 cells, we analyzed morphological nuclear changes using Hoechst 33258 staining. DNA fragmentation and loss of plasma membrane asymmetry are the most typical characteristics of apoptotic cell death. **Fig. 3** showed increased nuclear shrinkage, condensation and DNA fragmentation in abieslactone-treated cells compared to the untreated control. Cells pretreated with Z-VAD-FMK had no significant nuclear change after abieslactone treatment at a dose of 20 µM (**Fig. 3**). The data indicated that the caspase pathway was involved in abieslactone-induced HepG2 and SMMC7721 cell apoptosis.

### Abieslactone induces cell cycle arrest in G_1_ phase

**Figure 4:**
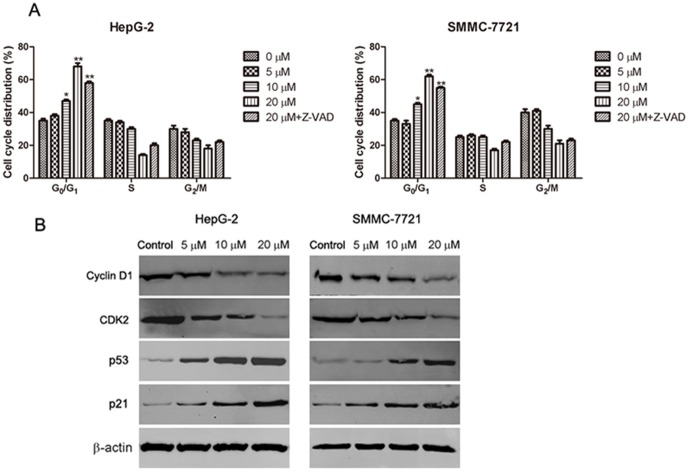
The effect of abieslactone on the cell cycle and the expression of cell cycle regulators in HepG2 and SMMC7721 cells. (A) Abieslactone treatment induced a dose dependent increase in the proportion of cells in the G_1_ phase and a decrease in cells in the S and G_2_ phases compared to the untreated control. Z-VAD-FMK treatment did not prevent cell cycle arrest following high-dose abieslactone treatment. The results are represented as the mean ± SEM for three independent experiments with similar results. *P<0.05; **P<0.01 *vs*. the untreated control. (B) Representative pictures for p53, p21 CDK2 and cyclin D1 protein expression by western blot analysis. β-actin was used as a control.

Cell cycle arrest is one of the major causes of cell death. To explore whether abieslactone-induced apoptosis was associated with cell cycle arrest, we examined cell cycle distribution in HepG2 and SMMC7721 cells using flow cytometry to analyze the DNA content in each cell cycle phase. As shown in **Fig. 4A**, abieslactone treatment induced a dose-dependent increase in the proportion of cells in the G_1_ phase and decrease in cells in the S and G_2_ phases compared to the untreated control. Furthermore, we used a general caspase inhibitor Z-VAD-FMK for the cell cycle analyses in HepG2 and SMMC7721 cells. Flow cytometric analysis showed that Z-VAD-FMK treatment did not prevent cell cycle arrest after high-dose abieslactone treatment (**Fig. 4A**), although abieslactone induced HepG2 and SMMC7721 cell apoptosis. The data indicate that abieslactone induced HepG2 and SMMC7721 cell death through cell cycle arrest in G_1_ phase and by the induction of apoptosis.

### Abieslactone induces the expression of cell cycle regulators

Having established that abieslactone induce cell-cycle arrest, we attempted to characterize, at the molecular level, the mechanisms by which this effect is achieved. The tumor suppressor protein, p53, regulates the cell cycle, and its target gene, p21, directly inhibits cyclin D1 and CDK2. This pathway results in cell cycle arrest in the G_1_ phase. To investigate the mechanism of abieslactone-induced cell cycle arrest in HepG2 and SMMC7721 cells, we analyzed p53, p21 cyclin D1 and CDK2 expression by western blotting. As illustrated in **Fig. 4B**, the expression levels of p53 and p21 were markedly increased, whereas cyclin D1 and CDK2 expression were significantly decreased in a dose dependent manner. These results revealed that abieslactone induced cell death in HepG2 and SMMC7721 cells through p53 activation, leading to cell cycle arrest in G_1_ phase.

### Abieslactone induces apoptosis in the mitochondria

**Figure 5:**
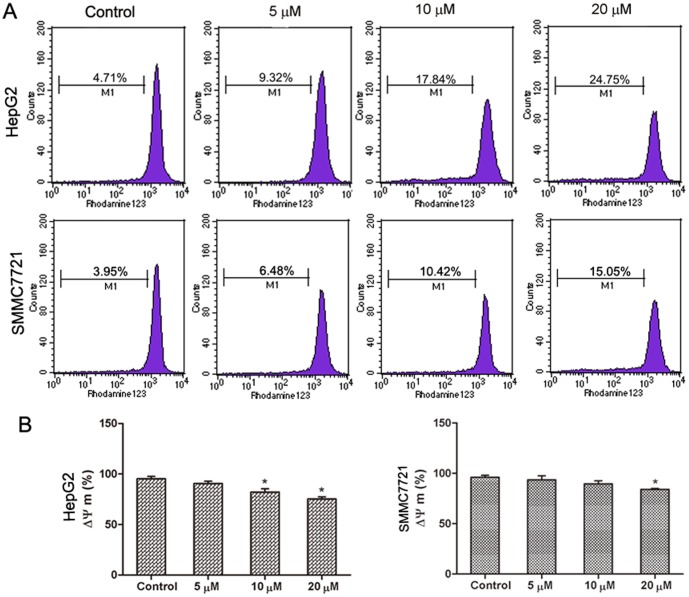
The effect of abieslactone on the MMP in HepG2 and SMMC7721 cells. (A) The MMP of HepG2 and SMMC7721 cells treated with abieslactone at different concentrations was analyzed by flow cytometry. (B) The loss of the MMP in HepG2 and SMMC7721 cells following abieslactone treatment in a dose dependent manner. The data are expressed as the means ± SEM for three independent experiments with similar results. *P<0.05 *vs*. the untreated control.

Another characteristic feature of apoptosis is depolarization of the mitochondrial membrane potential (MMP). To investigate whether abieslactone-induced cell apoptosis was associated with mitochondrial dysfunction, we analyzed MMP changes in HepG2 and SMMC7721 cells by staining with Rh123, a mitochondria-sensitive dye, and analyzing the cells by flow cytometry. Our results show that the MMP of HepG2 and SMMC7721 cells decreased significantly after treatment with abieslactone in a dose dependent manner (**Fig. 5**), suggesting that abieslactone induced cell apoptosis through the intrinsic pathway.

### Abieslactone induces cell apoptosis through a mitochondrial pathway

**Figure 6:**
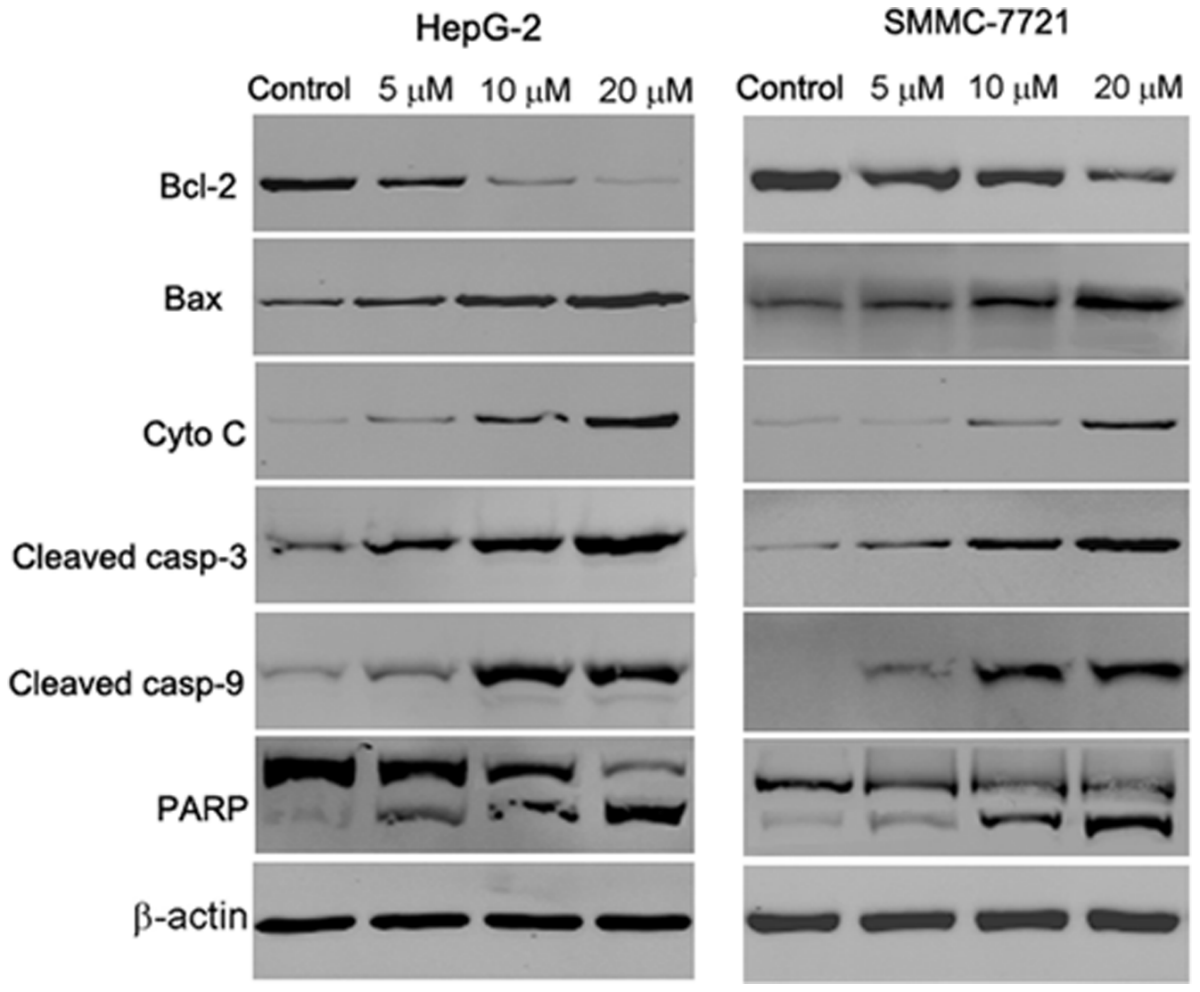
The effect of abieslactone on the expression of caspase-dependent mitochondrial apoptosis pathway proteins in HepG2 and SMMC7721 cells. Representative images of cytochrome c, Bax, Bcl-2, PARP, cleaved caspase 9 and cleaved caspase 3 protein expression detected by western blot. β-actin was used as a control.

Mitochondrial damage facilitates cytochrome c release from mitochondria into the cytoplasm and activates apoptotic factors (Bcl-2 family proteins), which leads to activation of the caspase cascade (apoptotic markers) and mitochondria-mediated apoptosis [28]. Activation of caspase cascade leading to PARP cleavage is regarded as a major pathway in apoptosis induction. To test whether abieslactone induces apoptosis through this mechanism in HepG2 and SMMC7721 cells, we examined the expression of cytochrome c, the pro-apoptotic protein Bax, the anti-apoptotic protein Bcl-2, caspase 3, caspase 9 and PARP by western blot analysis. In a dose dependent manner, abieslactone increased cytosolic cytochrome c, Bax, cleaved caspase 3 and cleaved caspase 9 expressions with a concomitant decrease in Bcl-2 expression compared to the untreated control (**Fig. 6**). Meanwhile, exposure of HepG2 and SMMC7721 cells to abieslactone also resulted in the cleavage of PARP fragment (**Fig. 6**), which is an endogenous substrate of activated caspase-3 and its cleavage is considered to be a hallmark of cell apoptosis. These results suggested that the mitochondria and Bcl-2 family members are involved in abieslactone-mediated cell apoptosis in HepG2 and SMMC7721 cells.

### ROS/Akt pathway is involved in the abieslactone-induced cell apoptosis in HepG2 cells but not in SMMC7721 cells

**Figure 7:**
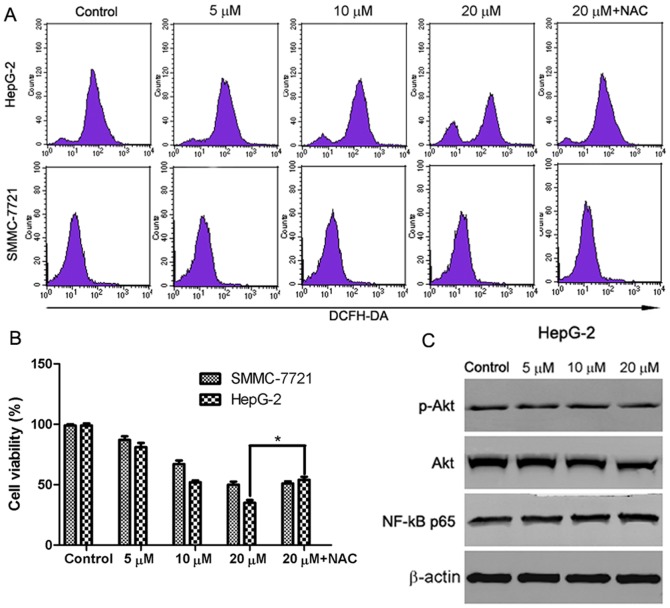
The involvement of ROS/Akt pathway in abieslactone-induced HepG2 cell apoptosis. (A) HepG2 and SMMC7721 cells were treated with different concentrations of abieslactone or ROS scavenger (NAC 10 mM), and then ROS was measured by DCF fluorescence analysis. The increased fluorescence of DCF was determined as the increased intracellular ROS accumulation. (B) HepG2 and SMMC7721 cells pretreated with 10 mM NAC for 1 h were incubated with 20 µM abieslactone for 24 h, and cell viability was determined by MTT assay. *P<0.05 *vs*. the untreated control. (C) The effect of abieslactone on the expression of p-Akt, Akt and NF-kB p65 proteins in HepG2 cells. β-actin was used as a control.

As ROS generation is important in mitochondria-mediated apoptosis, the effect of abieslactone on the generation of ROS was investigated. HepG2 and SMMC7721 cells were exposed to abieslactone at different concentrations for 24 h and analyzed for the accumulation of ROS by fluorescence microscopy following staining with DCFH-DA. As shown in **Fig. 7A**, treatment with abieslactone resulted in a significant increase in intracellular ROS in HepG2 cells. However, no significant increase in ROS levels was observed in SMMC7721 cells after 24 h of abieslactone treatment. Furthermore, we pretreated HepG2 cells with the ROS scavenger NAC at 10 mM for 1 h followed by abieslactone (20 µM) treatment for additional 24 h. The viability of HepG2 cells was partly rescued by ROS scavenger NAC (**Fig. 7B**). These results indicate that abieslactone may induce cell apoptosis by the generation of ROS in HepG2 cells.

Recent studies have demonstrated that downregulation of Akt and NF-kB activity is involved in mitochondria-mediated apoptosis and Akt and NF-kB signals are the downstream event of ROS generation in mitochondria-dependent apoptosis [20], [21]. We further investigated the effects of abieslactone on the expression of Akt and the p65/RelA subunit of NF-kB in HepG2 cells, which is necessary for the transactivation activity of NF-kB. Our results showed that abieslactone inhibited Akt activity in HepG2 cells; not only phosphorylated levels of Akt were reduced, but total Akt levels were decreased after abieslactone treatment for 24 h (**Fig. 7C**). However, abieslactone applied to HepG2 cells for 24 h did not change the expression of p65/RelA protein levels (**Fig. 7C**). Based on the results described above, abieslactone may induce HepG2 cell apoptosis *via* the ROS/Akt pathway.

## Discussion

Although traditional chemotherapy remains the mainstay in human cancer treatment, the response rates to most chemotherapeutic agents are low, and clinical improvement is marginal [27]. In addition, severe toxicities and drug resistance often occur, hindering the effective application of these agents. Considerable attention has been focused on identifying naturally occurring bioactive compounds and their derivatives capable of inhibiting or reversing the development of liver cancer. In the liver, the elimination of transformed cells via apoptosis induction is considered to be a crucial step for the treatment of liver cancer. We have been interested in discoverying new, effective, and safe drugs from natural products for cancer therapy. Previous studies have indicated that the *Abies* plants have ideal natural compounds for targeted treatment of many cancer cell lines [29]–[32]. Although some studies have demonstrated *in vitro* antitumor activity of triterpenoid lactones from *Abies* plants in various human tumor cell lines, their underlying mechanisms of action remain to be elucidated. Abieslactone is a triterpenoid lactone isolated from *A. faxoniana*, a folk medicine used to treat bronchitis, digestive disorders, and inflammation. Our data demonstrated that abieslactone inhibited HepG2 and SMMC7721 cell growth in a dose dependent manner. Interestingly, we found that the growth-inhibiting effect of abieslactone was tumor cell selective, as normal hepatic cell line (QSG7701) did not display a significant toxic effect *in vitro*.

Cell cycle and apoptosis are considered to be two major regulatory mechanisms for cell growth. When specific checkpoints during the cell cycle are arrested, apoptotic cell death occurs [33]. Moreover, many chemotherapeutic agents cause cell cycle arrest through microtubule damage and have been proven to be clinically effective for treating cancer [34]. Flow cytometric studies showed that the growth inhibition induced by abieslactone occurs through the cell cycle arrest of HepG2 and SMMC7721 cells in the G_1_ phase. The G_1_ to S cell cycle progression is controlled by several cyclin-dependent kinase (CDK) complexes, the activities of which are dependent on the balance of cyclins and cyclin-dependent kinase inhibitors (CKIs). p53, the most extensively studied tumor suppressor, mediates a variety of anti-proliferative processes through cell cycle checkpoints, DNA repair and apoptosis [35]. Previous reports have found that p21, the target of p53, is one of the major CKIs, which directly inhibit the activity of CDKs, thereby leading to cell cycle arrest in the G_1_ phase [36]–[38]. Upregulation of p21 and p53 expression may inhibit cyclin/CDK complexes, thus leading to cell G_1_ cycle arrest. To gain insight into the molecular mechanisms of abieslactone-induced G_1_ arrest in HepG2 and SMMC7721 cells, we examined p53, p21 cyclin D and CDK2 expression. Western blot analysis revealed that abieslactone treatment downregulated CDK2 and cyclin D expression and reversed the reduction in p53 and p21 expression, which enhances the formation of heterotrimeric complexes with the CDKs and cyclins, thereby leading to cell cycle arrest in the G_1_ phase. The results indicated that one of the mechanisms of abieslactone in the suppression of HepG2 and SMMC7721 cell growth may be the inhibition of cell G_1_ cycle progression through a p53-dependent pathway.

G_1_ phase arrest of cell cycle regulation provides an opportunity for cells to follow the apoptotic pathway. Although it was shown that abieslactone suppresses HepG2 and SMMC7721 cell growth through the induction of G_1_ phase arrest, we cannot be sure that they are the only factors reducing cell growth. To determine whether abieslactone-induced inhibition of HepG2 and SMMC7721 cell growth is dependent on apoptosis, we performed flow cytometric analysis of apoptosis after treating with abieslactone. The data showed that abieslactone induced HepG2 and SMMC7721 cell apoptosis in a dose dependent manner. Furthermore, after preincubation with Z-VAD-FMK, apoptosis was greatly attenuated, indicating that apoptosis occurs via a caspase-dependent pathway. Moreover, we also found that inhibition of caspase activation did not prevent cell cycle arrest, suggesting that abieslactone inhibited HepG2 and SMMC7721 cell growth through either cell cycle arrest or apoptosis induction.

Apoptosis occurs through two main pathways: death receptor pathway and intracellular mitochondrial pathway. The mitochondrial pathway is a key signaling pathway in apoptosis induction. The Bcl-2 family proteins are usually involved in mitochondrial apoptotic signal pathway. The Bcl-2 protein family, whose members may be anti-apoptotic and pro-apoptotic proteins, regulates cell death by controlling mitochondrial membrane permeability during apoptosis [39]. Bcl-2 is a potent antiapoptotic factor, whereas Bax, an antagonist of Bcl-2, is inserted into the outer membrane of the mitochondria, allowing for the release of cytochrome c and activating caspase 9. Caspase-3 is activated by proteolytic cleavage of caspase 9 and is a key apoptotic executive caspase. Activation of the caspase cascade leading to PARP cleavage is regarded as a major factor in the apoptotic signaling pathway. Involvement of the mitochondrial pathway in cell apoptosis was first confirmed by observing changes in MMP and Bcl-2 and Bax protein expression. Mitochondrial dysfunction often involves MMP loss and cytochrome c release from mitochondria into the cytosol. We found that abieslactone decreased the MMP, causing cytochrome c release from the mitochondria into the cytoplasm. Western blot analysis indicated that abieslactone resulted in significant decrease in the level of Bcl-2 protein and increased Bax protein, thus shifting the Bax/Bcl-2 ratio in favor of apoptosis. Moreover, abieslactone increased caspase 3 and caspase 9 as well as cleaved PARP expression in a dose dependent manner. Furthermore, pretreatment with Z-VAD-FMK resulted in a significant decrease in apoptosis, indicating that caspase was involved in abieslactone-induced apoptosis. Therefore, we concluded that abieslactone induced caspase-dependent apoptosis via the mitochondrial pathway.

ROS generation is also an important mediator of many anti-cancer agents. Previous studies have shown that ROS produced by chemotherapy are essential for inducing apoptosis in some kinds of cancer [40]. ROS, which were predominantly produced in the mitochondria, if excessive, may lead to the free radical attack of membrane phospholipids and loss of MMP, which causes the release of apoptosis-inducing factors that activate caspase cascades and cause nuclear condensation [41]. ROS generation is viewed as one of the main mechanisms of mitochondria-dependent apoptosis [42]. The results showed that the accumulation of ROS was detected in HepG2 cells when treated with abieslactone. However, no significant increase in ROS levels was observed in SMMC7721 cells after abieslactone treatment. NAC is a potent antioxidant that may inhibit oxidative stress by directly scavenging ROS. To identify the role of ROS in abieslactone-induced apoptosis in HepG2 cells, cell death was measured following treatment of abieslactone with NAC. Abieslactone (20 µM) significantly increased HepG2 cell death, whereas removing intracellular ROS by NAC partly inhibited abieslactone-induced cell death. These results revealed that abieslactone-induced ROS accumulation was involved in HepG2 cell apoptosis.

Akt is a critical kinase involved in cell signal transduction cascades promoting cell survival by inhibiting apoptosis through its ability to phosphorylate and inactivate several proapoptotic proteins [43]. It has been reported that some clinical chemotherapeutic agents could induce cancer cell apoptosis by regulating Akt signal pathway [44]. NF-kB, a pro-inflammatory nuclear transcription factor, regulates genes important for tumor invasion, metastasis, angiogenesis, and chemoresistance [45]. Exposure of cancer cells to anticancer drugs can induce the activation of the NF-kB pathway, leading to the expression of anti-apoptotic genesand resistance to apoptosis. Recent studies have shown that downregulation of Akt and NF-kB activity is involved in mitochondria-mediated apoptosis, and Akt and NF-kB signals appear to be a downstream regulator of ROS generation. To determine if abieslactone-induced apoptosis was mediated by regulating Akt and NF-kB activity, we treated HepG2 cells with increasing concentrations of abieslactone. Our results show that abieslactone inhibited total and phosphorylated Akt activities. However, abieslactone applied to HepG2 cells did not change p65/RelA protein levels. Thus, we concluded that abieslactone induced HepG2 cell apoptosis via the ROS/Akt pathway.

**Figure 8:**
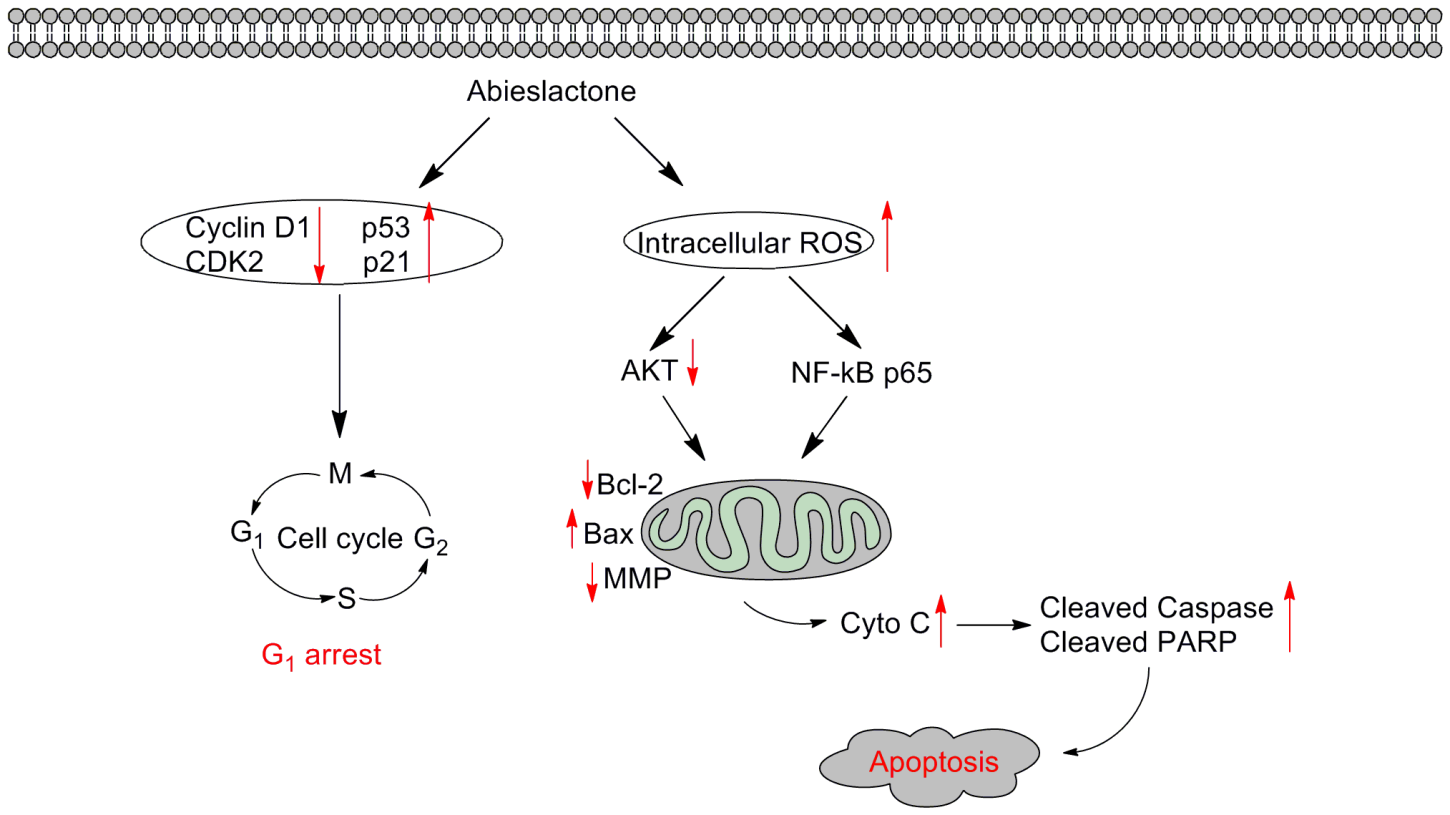
Schematic form of the proposed mechanisms for abieslactone-induced G_1_ arrest and apoptosis in HepG2 cells.

In conclusion, our present studies show that abieslactone exerted an inhibitory effect on hepatoma cells by arresting the cell cycle in the G_1_ phase and inducing apoptosis (**Fig. 8**). G_1_ phase arrest was found to be associated with the upregulation of p53 and p21 and the downregulation of CDK2 and cyclin D. Furthermore, the induction of apoptosis was associated with activation of caspase cascades, disruption of MMP, release of cytochrome c and modulation of Bcl-2 family proteins. In addition, ROS accumulation and inactivation of Akt kinase were involved in abieslactone-induced HepG2 cell apoptosis.
